# Risk of urinary bladder cancer: a case-control analysis of industry and occupation

**DOI:** 10.1186/1471-2407-9-443

**Published:** 2009-12-15

**Authors:** Adrian Cassidy, Wei Wang, Xifeng Wu, Jie Lin

**Affiliations:** 1 Department of Epidemiology, The University of Texas M.D. Anderson Cancer Center, Houston, Texas, USA

## Abstract

**Background:**

Uncertainty remains about urinary bladder cancer (UBC) risk for many occupations. Here, we investigate the association between occupation, industry and UBC.

**Methods:**

Lifetime occupational history was collected by in-person interview for 604 newly diagnosed UBC patients and 604 cancer-free controls. Each job title was assigned a two-digit industry code and a three-digit occupation code. Odds ratios (ORs) for UBC associated with ever being employed in an industry or occupation were calculated by unconditional logistic regression adjusting for age, gender and smoking status. We also examined UBC risk by duration of employment (>0 to <10, ≥10 years) in industry or occupation.

**Results:**

Significantly increased risk of UBC was observed among waiters and bartenders (OR 2.87; 95% CI 1.05 to 7.72) and occupations related to medicine and health (OR 2.17; 95% CI 1.21 to 3.92), agricultural production, livestock and animal specialties (OR 1.90; 95% CI 1.03 to 3.49), electrical assembly, installation and repair (OR 1.69; 95% CI 1.07 to 2.65), communications (OR 1.74; 95% CI 1.00 to 3.01), and health services (OR 1.58; 95% CI 1.02 to 2.44). For these occupations we also observed a significant excess risk of UBC for long-term work (i.e. ≥10 years), with the exception of waiters and bartenders. Employment for 10 years or more was associated with increased risk of UBC in general farmers (OR 9.58; 95% CI 2.18 to 42.05), agricultural production of crops (OR 3.36; 95% CI 1.10 to 10.27), occupations related to bench working (OR 4.76; 95% CI 1.74 to 13.01), agricultural, fishery, forestry & related (OR 4.58; 95% CI 1.97 to 10.65), transportation equipment (OR 2.68; 95% CI 1.03 to 6.97), and structural work (OR 1.85; 95% CI 1.16 to 2.95).

**Conclusions:**

This study provides evidence of increased risk of UBC for occupations that were previously reported as at-risk. Workers in several occupation and industry groups have a significantly higher risk of UBC, particularly when duration of employment is 10 years or more.

## Background

Urinary bladder cancer (UBC) ranks the fourth most common cancer in men and ninth in women in the United States, accounting for an estimated 70,980 new cases in 2009[1]. Numerous studies have demonstrated that the primary modifiable risk factors for UBC are cigarette smoking and occupational exposure to carcinogens. An updated review of the literature estimates that between 5 and 25% of all UBC cases are attributable to workplace exposures[2]. The variance in attributable fraction suggests that, despite numerous studies investigating the association between UBC risk and occupational exposures, results have been inconsistent. To date, only a few occupations and industries, such as those exposed to aromatic amines, have been unequivocally associated with increased risk of UBC.

Previous reported high-risk occupation/industry groups include dyestuff workers, rubber workers, painters, printers, metal workers, textile workers, truck drivers, chemical workers and employment in industries involving leather, petrochemical production, plastics production and hairdressing. [3-13] Excess risk of UBC has also been reported among health service workers and farmers[3,4,6,14-16]. However, uncertainty remains about UBC risk for many occupations[17]. Using data from a large on-going case-control study of UBC in Houston, Texas, we sought to investigate the association between occupation, industry and UBC, and to identify general patterns and trends in UBC risk in the study population.

## Methods

Urinary bladder cancer patients were enrolled from an on-going case-control study of UBC at M.D. Anderson Cancer Center, Houston, Texas, which began recruitment in 1999. The procedures for patient recruitment and eligibility criteria have been described elsewhere[18]. Briefly, UBC patients were recruited from the University of Texas M.D. Anderson Cancer Center and Baylor College of Medicine. Patients were defined as newly diagnosed and histopathologically confirmed UBC patients who had not previously received chemotherapy or radiotherapy. There were no recruitment restrictions on age, gender, ethnicity or cancer-stage. The controls were recruited in collaboration with the Kelsey-Seybold clinics, the largest private multi-specialty physician group consisting of more than 23 clinics and over 300 physicians in the Houston metropolitan area. The majority of controls visited the clinics for annual health check-ups. Controls were frequency matched to case by age (± 5 years), gender and ethnicity. Controls had no prior history of cancer (except non-melanoma skin cancer). After obtaining written, informed consent from the patients and controls, trained M.D. Anderson Cancer Center staff interviewers administered risk factor questionnaires that collected data on demographic characteristics, lifestyle factors, family history of cancer and occupational history. Participation rates are approximately 92% for patients and 75% for controls.

For occupational history, subjects were asked to describe all occupations that they had held for at least 1 year. To assist in assigning the correct occupational codes, detailed information was requested on job title, major duties on the job, equipment/materials/chemicals used while performing the job, and the type of work that the employer company specialized in. Participants were also asked to report the duration of employment for each job. The period of time to complete the interview was approximately 45 minutes. Human subject approval was obtained from the institutional review boards (IRB) of M.D. Anderson Cancer Center, Baylor College of Medicine and Kelsey-Seybold Clinics.

For each job title, occupation and industry categories were coded according to the Dictionary of Occupational Titles (OCC)[19] and Standard Industrial Classification (SIC) [20], respectively. Specifically, each job title was assigned a two-digit SIC code, and a three-digit OCC code.

Pearson's χ^2 ^test was used to test the differences between patients and controls in terms of gender and smoking status. Student's *t *test was used to test differences in age and pack-years of cigarette smoking between patients and controls. We used unconditional logistic regression to calculate odds ratios (OR) and corresponding 95 percent confidence intervals (CI) for ever being employed in an industry or occupation, compared to never being employed in that industry or occupation. We also estimated the risk of UBC stratified by the duration of employment (>0 to <10 years, ≥10 years) in industry and occupation groups. Ten years was used as the cut-off for duration because of small numbers of patients and controls in more tightly defined categories. Due to the small sample size of minority groups, we restricted analyses to Caucasians. ORs were adjusted for age, sex and smoking status. All statistical tests were two-sided with a significance level of 0.05. Data analysis was performed using Intercooled Stata 8.2 (Stata Corporation, College Station, Texas).

## Results

The study population consisted of 604 patients with UBC and 604 cancer-free controls. The distribution of patients and controls in terms of age, gender, and smoking status is presented in Table 1. The distribution of smoking status differed significantly in patients and controls. Specifically, 73% of patients were ever smokers as compared to 53.3% of controls. UBC patients were also heavier smokers (mean pack-years, 43.1) than the controls (mean pack-years, 28.8) (*P *< 0.0001).

**Table 1:** Characteristics of the Study Population

	Case	Control	
Characteristic	(N = 604)	(N = 604)	*P *Value
Gender, N (%)			
Male	464 (76.8)	464 (76.8)	
Female	140 (23.2)	140 (23.2)	0.99
			
Smoking status, N (%)			
Never	163 (27.0)	282 (46.7)	
Former	279 (46.2)	277 (45.9)	
Current	162 (26.8)	45 (7.5)	<0.0001
Ever	441 (73.0)	322 (53.3)	<0.0001
			
Pack-year, mean (SD)	43.14 (30.5)	28.81 (27.7)	<0.0001
Age, mean (SD)	63.64 (11.0)	63.02 (10.5)	0.99

Increased risk of UBC was observed for occupations in medicine and health (OR 2.17; 95% CI 1.21 to 3.92), and for waiters and bartenders (OR 2.84; 95% CI 1.05 to 7.72). Borderline significant associations were observed for occupations in electrical assembly, installation and repair (OR 1.69; 95% CI 0.99 to 2.89), law and jurisprudence (OR 1.89; 95% CI 0.90 to 3.95), and food and beverage preparation, and service (OR 1.82; 95% CI 0.91 to 3.66). Non-significant elevated risk of UBC was also observed for occupations in motor freight (OR 1.43; 95% CI 0.81 to 2.53), general farming (OR 1.51; 95% CI 0.79 to 2.89), and structural work (OR 2.21; 95% CI 0.80 to 6.11) (Table 2).

**Table 2:** Risk of urinary bladder cancer for occupation group, overall and by duration of employment*

	Overall			>0 to <10 years			≥ 10 years		
			
Occupation Group	Case	Control	OR (95%CI)	Case	Control	OR (95%CI)	Case	Control	OR (95%CI)
Occupations in medicine and health	35	20	2.17 (1.21 to 3.92)	7	9	1.08 (0.39 to 2.98)	28	11	3.07 (1.47 to 6.40)
Electrical assembly, installation, and repair	41	25	1.69 (0.99 to 2.89)	16	20	0.95 (0.47 to 1.90)	25	5	4.37 (1.62 to 11.7)
General farming occupations	26	17	1.51 (0.79 to 2.89)	7	15	0.43 (0.17 to 1.13)	19	2	9.58 (2.18 to 42.1)
Farmer (plant farming, miscellaneous agricultural and related occupations)	39	26	1.39 (0.81 to 2.38)	13	23	0.41 (0.18 to 0.93)	26	3	7.99 (2.36 to 27.1)
Agricultural, fishery, forestry and related occupations	50	34	1.39 (0.86 to 2.23)	17	27	0.56 (0.29 to 1.09)	33	7	4.58 (1.97 to 10.7)
Waiters, bartenders	17	6	2.84 (1.05 to 7.72)	12	4	3.52 (1.06 to 11.65)	5	2	1.64 (0.28 to 9.74)
Structural work occupations	116	89	1.25 (0.90 to 1.74)	50	57	0.90 (0.59 to 1.39)	66	32	1.85 (1.16 to 2.95)
Bench work occupations	39	25	1.44 (0.84 to 2.47)	19	20	0.71 (0.36 to 1.41)	20	5	4.76 (1.74 to 13.01)
Fabrication and repair of textile, leather, and related products	7	0	---	5	0	---	2	0	---
Occupations in law and jurisprudence	20	13	1.89 (0.90 to 3.95)	4	3	1.35 (0.29 to 6.34)	16	10	2.06 (0.89 to 4.75)
Food and beverage preparation and service	26	14	1.82 (0.91 to 3.66)	17	10	1.85 (0.81 to 4.25)	9	4	1.75 (0.50 to 6.18)
Occupations in entertainment and recreation	10	6	1.25 (0.43 to 3.65)	7	5	0.98 (0.29 to 3.35)	3	1	2.64 (0.26 to 26.8)
Production and stock clerks and related occupations	17	13	1.18 (0.55 to 2.53)	10	9	0.88 (0.34 to 2.31)	7	4	1.90 (0.54 to 6.70)
Amusement and recreation service occupations	6	3	1.92 (0.45 to 8.15)	4	3	1.43 (0.30 to 6.76)	2	0	---
Apparel and furnishings service occupations	5	3	1.36 (0.31 to 6.01)	3	2	1.09 (0.16 to 7.25)	2	1	1.89 (0.17 to 21.0)
Janitor	6	3	1.71 (0.39 to 7.50)	4	2	1.63 (0.27 to 9.79)	2	1	1.88 (0.14 to 25.1)
Occupations in processing of food, tobacco, and related products	11	9	1.12 (0.43 to 2.87)	4	4	0.76 (0.17 to 3.44)	9	5	1.67 (0.52 to 5.38)
Mechanics and machinery repairers	43	35	1.09 (0.66 to 1.78)	24	22	0.94 (0.50 to 1.77)	19	13	1.34 (0.63 to 2.87)
Auto mechanics	23	19	1.05 (0.54 to 2.04)	13	9	1.26 (0.50 to 3.14)	10	10	0.87 (0.34 to 2.23)
Occupations in fabrication and repair of electrical equipment	10	7	1.39 (0.51 to 3.81)	8	6	1.31 (0.43 to 3.98)	2	1	1.79 (0.16 to 20.0)
Occupations in fabrication and repair of products made from assorted materials	5	2	2.09 (0.38 to 11.42)	3	2	1.34 (0.21 to 8.63)	2	0	---
Fabrication and repair of wood products	4	1	6.42 (0.70 to 58.93)	0	1	---	4	0	---
Occupations in metal fabricating, not elsewhere classified	10	8	1.13 (0.42 to 3.07)	5	7	0.54 (0.16 to 1.87)	5	1	5.99 (0.67 to 53.8)
Excavating, grading, paving, and related occupations	9	5	1.45 (0.46 to 4.53)	4	1	4.11 (0.44 to 38.28)	5	4	0.98 (0.26 to 3.67)
Structural work occupations, not elsewhere classified	13	6	2.21 (0.80 to 6.11)	11	5	2.20 (0.72 to 6.75)	2	1	2.24 (0.20 to 25.0)
Motor freight occupations	37	22	1.43 (0.81 to 2.53)	22	13	1.49 (0.72 to 3.11)	15	9	1.30 (0.57 to 3.01)
Automobile drivers	40	30	1.13 (0.68 to 1.89)	25	17	1.30 (0.67 to 2.51)	15	13	0.94 (0.44 to 2.02)
Occupations in extraction of minerals	17	11	1.48 (0.66 to 3.32)	10	10	1.09 (0.44 to 2.75)	7	1	5.00 (0.56 to 44.4)

*Adjusted for age, gender and smoking status

Next, we performed analysis stratified by duration of employment. More than 10 years employment in plant farming and miscellaneous agricultural was associated with an 8-fold higher OR for UBC (95% CI 2.36 to 27.07), whilst a reduced risk of UBC was observed for less than 10 years employment (OR 0.41; 95% CI 0.18 to 0.93). Employment in general farming for more than 10 years was associated with 9.58-fold (95% CI 2.18 to 42.05) increased risk of UBC. Significantly elevated risk of UBC was also evident among those employed for 10 years or more in occupations related to electrical assembly, installation and repair (OR 4.37; 95% CI 1.62 to 11.77), bench work (OR 4.76; 95% CI 1.74 to 13.01), medicine and healthcare (OR 3.07; 95% CI 1.47 to 6.40), and structural work (OR 1.85; 95% CI 1.16 to 2.95).

Industry-specific analysis identified increased risk of UBC among those employed in agriculture production livestock and animal specialties (OR 1.90; 95% CI 1.03 to 3.49), communications (OR 1.74; 95% CI 1.00 to 3.01) and health service (OR 1.58; 95% CI 1.02 to 2.44). Significantly reduced risk of UBC was observed for individuals engaged in the primary metal (OR 0.48; 95% CI 0.24 to 0.94), and petroleum refining and related industries (OR 0.65; 95% CI 0.43 to 1.00). We also observed a reduced risk of UBC among those employed for less than 10 years in the industry group, building construction, general contractors and operative builders (OR 0.37; 95% CI 0.18 to 0.74). Employment for 10 years or more in agricultural production livestock and animal specialties conferred a significant elevated risk of UBC (OR 6.18; 95% CI 2.09 to 18.29). An increased risk of UBC was also observed among those employed for more than 10 years in industries related to agricultural production of crops (OR 3.36; 95% CI 1.10 to 10.27), transportation equipment (OR 2.68; 95% CI 1.03 to 6.97), and health services (OR 2.31; 95% CI 1.33 to 4.01) (Table 3).

**Table 3:** Risk of urinary bladder cancer for industry group, overall and by duration of employment*

	Overall			>0 to <10 years			≥ 10 years		
			
Industry Group	Case	Control	OR (95%CI)	Case	Control	OR (95%CI)	Case	Control	OR (95%CI)
Agricultural production crops	27	20	1.09 (0.59 to 2.01)	9	16	0.48 (0.20 to 1.14)	18	4	3.36 (1.10 to 10.3)
Agriculture production livestock and animal specialties	37	17	1.90 (1.03 to 3.49)	12	13	0.68 (0.30 to 1.58/)	25	4	6.18 (2.09 to 18.3)
Transportation equipment	40	25	1.39 (0.81 to 2.37)	21	19	0.99 (0.51 to 1.90)	19	6	2.68 (1.03 to 6.97)
Communications	39	23	1.74 (1.00 to 3.01)	13	11	1.49 (0.64 to 3.45)	26	12	2.01 (0.98 to 4.10)
Health services	59	50	1.58 (1.02 to 2.44)	17	24	0.84 (0.43 to 1.67)	42	26	2.31 (1.33 to 4.01)
Agriculture services	13	7	1.54 (0.59 to 4.06)	8	3	2.67 (0.67 to 10.65)	5	4	0.83 (0.21 to 3.29)
Fishing, hunting, and trapping	6	1	4.16 (0.47 to 36.62)	3	1	1.41 (0.13 to 15.37)	3	0	---
Coal mining	3	1	2.69 (0.22 to 32.30)	1	1	0.64 (0.03 to 15.76)	2	0	---
Oil and gas extraction	14	7	1.75 (0.67 to 4.59)	11	6	1.52 (0.53 to 4.36)	3	1	3.28 (0.32 to 34.2)
Mining and quarrying of nonmetallic minerals, except fuels	3	2	1.40 (0.23 to 8.51)	1	1	1.11 (0.07 to 17.94)	2	1	1.65 (0.15 to 18.5)
Building construction, general contractors and operative builders	42	44	0.73 (0.46 to 1.17)	14	26	0.37 (0.18 to 0.74)	28	18	1.26 (0.67 to 2.37)
Petroleum refining and related industries	42	67	0.65 (0.43 to 1.00)	13	18	0.75 (0.35 to 1.58)	29	49	0.62 (0.38 to 1.02)
Textile mill products	10	4	3.04 (0.79 to 11.72)	4	2	1.86 (0.31 to 11.26)	6	1	5.31 (0.61 to 46.4)
Furniture and fixtures	7	3	2.23 (0.54 to 9.31)	3	1	1.52 (0.14 to 15.93)	4	2	2.70 (0.47 to 15.6)
Paper and allied products	13	7	1.83 (0.70 to 4.79)	7	6	1.11 (0.35 to 3.54)	6	1	6.06 (0.70 to 52.2)
Printing, publishing, and allied industries	15	9	1.37 (0.56 to 3.30)	9	5	1.49 (0.47 to 4.75)	6	4	1.21 (0.31 to 4.68)
Rubber and miscellaneous plastics products	18	14	1.03 (0.49 to 2.16)	7	3	1.64 (0.40 to 6.71)	11	11	0.89 (0.37 to 2.13)
Leather and leather products	6	0	---	5	0	---	1	0	---
Stone, clay, glass, and concrete products	13	9	1.50 (0.61 to 3.71)	6	5	1.21 (036 to 4.09)	7	4	1.46 (0.42 to 5.10)
Primary metal industries	17	24	0.48 (0.24 to 0.94)	10	16	0.45 (0.19 to 1.03)	7	8	0.62 (0.22 to 1.77)
Railroad transportation	13	7	1.46 (0.56 to 3.75)	7	4	1.39 (0.40 to 4.87)	6	3	1.54 (0.37 to 6.42)
Local and suburban transit and interurban highway passenger transport	8	5	1.42 (0.44 to 4.56)	7	3	1.96 (0.48 to 8.04)	1	2	0.57 (0.05 to 6.42)
Motor freight transportation and warehousing	36	22	1.17 (0.66 to 2.07)	18	11	1.18 (0.54 to 2.60)	18	11	1.16 (0.52 to 2.58)
Water transportation	8	5	1.13 (0.35 to 3.70)	6	5	0.76 (0.21 to 2.72)	2	0	---
Automotive dealers and gasoline service stations	37	31	0.98 (0.58 to 1.64)	22	23	0.81 (0.43 to 1.53)	15	8	1.47 (0.59 to 3.63)
Home furniture, furnishings, and equipment stores	17	9	1.54 (0.66 to 3.59)	11	7	1.45 (0.54 to 3.86)	6	2	2.25 (0.44 to 11.5)
Automotive repair, services, and parking	19	18	0.80 (0.40 to 1.61)	8	10	0.65 (0.24 to 1.73)	11	8	0.99 (0.37 to 2.67)
Educational services	104	107	1.21 (0.89 to 1.67)	50	50	1.16 (0.75 to 1.80)	54	57	1.31 (0.86 to 1.98)
Administration of environmental quality and housing programs	11	6	2.00 (0.72 to 5.56)	6	5	1.43 (0.43 to 4.79)	5	1	4.70 (0.53 to 41.7)

*Adjusted for age gender and smoking status

## Discussion

We observed a significantly elevated risk of UBC among waiters and bartenders, occupations related to electrical assembly, installation, and repair, workers in agriculture production livestock and animal specialties, agricultural production of crops, plant farmers, and employees in medicine and health services. Employment in primary metal and petroleum refining and related industries conferred a reduced risk of UBC, as did less than 10 years employment in the building construction industry and general farming. For the most part however, the current study demonstrated an increased risk of UBC with duration of employment for occupations and industries such as agricultural production livestock and animal specialties, health services, communications, medicine and health, electrical assembly installation and repair, structural work and bench work.

Whilst general farming occupations were not associated with an elevated risk of UBC, a nine-fold excess risk of UBC was observed for long-term employment (≥10 years) in general farming occupations. Similarly, overall employment in the agricultural production of crops industry was not associated with an excess risk of UBC, but those employed for 10 years or more had a 3.36-fold (95% CI 1.10 to 10.27) increased risk of UBC. However, less than 10 years employment in plant farming and miscellaneous agricultural occupations conferred a significantly reduced risk of UBC (OR 0.41; 95% CI 0.18 to 0.93). Associations between farming and bladder cancer risk have been reported previously, but findings are inconsistent[4,5,13-15,21-26]. For instance, 't Mannetje and colleagues reported significantly elevated risk of UBC for field, crop and vegetable farm workers employed for 25 years or more[14]. However, a more recent study reported a reduced risk of UBC among male farmers and those who worked in agricultural industries - even after accounting for duration of employment[13]. It is generally accepted that agricultural workers are potentially exposed to carcinogenic agents such as pesticides, solvents, inorganic dusts, and ultraviolet light during the course of their work. Although we cannot determine which exposure may have contributed to the risk observed in farming, and farming-related occupations and industries, our results suggest that UBC risks were pronounced among individuals employed for 10 years or more.

Consistent with previous findings, we observed an increased risk of UBC for workers in medicine and health occupations, and in the health services industry. Colt and colleagues reported an increased risk of UBC and a significant trend with duration of employment among female health service workers (OR 4.1; 95% CI 1.6 to 10.7; *P*_trend _= 0.014)[6]. Increased standardized incidence rates for UBC among health care workers in Sweden have also been reported, although the authors suggested that increased awareness to health conditions, knowledge related to symptoms, and easy access to screening and treatment among health care workers may have resulted in an earlier diagnosis of diseases than the general population[27]. On the other hand, sedentary work or frequency of urination may be a risk factor for UBC. Physical inactivity might lead to urinary retention and a higher urinary pressure, resulting in more intense and prolonged contact between urine borne carcinogenic agents and the sensitive basal cells of the distended urothelium[27]. The elevated risk of UBC observed in the communications industry, and for occupations in law and jurisprudence may also be due the sedentary nature of the work or frequency of urination, although this information was not collected here. Capturing data on urination frequency may help future studies elucidate the potential risk of UBC in sedentary occupations.

The increased risk of UBC observed among waiters and bartenders is more likely to be attributed to factors indirectly related to the job such as environmental tobacco smoke, and reduced frequency of urination and lower water intake[28,29]. Previous studies have suggested that total and specific fluid consumption is associated with an increased risk of UBC[28,30-33]. Increased fluid intake has been found to be a protective factor in some studies and it is hypothesized that increased fluid intake reduces the exposure of urothelium cells to carcinogenic compounds and metabolites[34]. Conversely, increased intake of fluid has been found to increase the risk of UBC potentially because it enhances exposure to carcinogens present in the fluid[33]. Fluid intake was not available in the current analysis: future studies should also incorporate measures of specific types of fluid intake.

Our study suggests an increased risk of UBC among workers employed in electrical assembly, installation, and repair, and communication industries, and for long-term employees (≥10 years) in the transportation equipment industry. Kogevinas and colleagues showed an elevated risk of UBC among electrical workers (OR 3.99; 95% CI 1.10 to 14.51) after combining data from 11 case-control studies in six European countries[4]. Band and colleagues reported a significant elevated risk of bladder cancer among workers engaged in occupations and industries relevant to electrical/electronic equipment, communications power and telecommunications transmission lines, possibly as a result of exposure to electromagnetic fields[35]. The same study reported a significantly increased risk of UBC for workers ever employed in the transportation equipment industry (OR 1.23; 95% CI 1.04 to 1.46)[35].

More than 10 years employment in occupations related to structural work, and bench work was associated with a significantly increased risk of UBC. It is important to note that the aggregation of occupations with very different exposures may obscure associations. For instance, structural work includes job titles such as painter and stone mason. Nevertheless, previous findings support an increased risk of UBC among painters and individuals who worked with varnish[36]. Also, exposures that may be prevalent in occupations related to bench work and structural work such as coal tar pitch volatiles, benzene soluble materials, and benzo(a)pyrene, have previously been associated with an elevated risk of UBC[37,38].

This study has several limitations. First, similar to other case-control studies of occupation, small sample size in many occupations limits statistical power to detect an association. Stratification by duration of employment accentuated small numbers of patients and controls in each occupation or industry category, which lead to wide confidence intervals. Also, some of the observed associations may have occurred by chance because of multiple comparisons. Second, job and industry titles are only crude surrogates for exposure. Any bias is likely to result in an attenuation of the association by grouping high and low exposed workers within one broad category. Also it is important to note that even within job and industry titles, exposures may differ in terms of exposure type, duration and intensity. Therefore, the findings from this study should be interpreted cautiously.

Examination of UBC risk by duration of employment is a particular strength of this study. In some occupations and industries, risk of developing UBC after less than 10 years employment was not significant, but became significant when this was extended to more than 10 years employment. This may suggest an exposure-response relationship, and highlights the advantage of using lifetime occupational histories over recording the current or last job. We were unable to adequately examine duration of employment by industry and occupation among women due to small numbers. We did not control for potential confounders such as residential risk to specific chemicals previously reported to be associated with bladder cancer; however occupational exposure levels are likely to be much higher. Nor did we control for other potential confounders such as family history, socioeconomic status, and alcohol use because of the potential to create small numbers within categories.

## Conclusion

Our study corroborates previous findings that agricultural occupations, occupations in medicine and health, electrical assembling installing and repairing occupations, and occupation as waiters and bartenders are associated with increased risk of UBC, particularly when duration of employment is 10 years or more. Our study also provides evidence of increased risk of UBC for occupations that were previously reported as at-risk occupations, such as transportation equipment, bench work and structural work. Further investigations are warranted to explore these putative associations.

## Competing interests

The authors declare that they have no competing interests.

## Authors' contributions

XW and JL designed the study, and collected the data. WW and AC analyzed the data, and drafted the manuscript. All authors read, gave comments, and approved the final version of the manuscript. WW, XW, JL and AC had full access to all of the data in the study and take responsibility for the integrity of the data and the accuracy of the data analysis. All authors read and approved the final manuscript.

## Pre-publication history

The pre-publication history for this paper can be accessed here:

http://www.biomedcentral.com/1471-2407/9/443/prepub
